# The study of the effectiveness of design-based engineering learning: the mediating role of cognitive engagement and the moderating role of modes of engagement

**DOI:** 10.3389/fpsyg.2023.1151610

**Published:** 2023-05-26

**Authors:** Lina Wei, Wei Zhang, Chenhua Lin

**Affiliations:** ^1^Institute of Medical Education/National Center for Health Professions Education Development, Peking University, Beijing, China; ^2^School of Education, Peking University, Beijing, China; ^3^School of Public Affairs, Zhejiang University, Hangzhou, China; ^4^Institute of China's Science, Technology and Education Policy, Zhejiang University, Hangzhou, China

**Keywords:** engineering education, design-based engineering learning, learning outcomes, cognitive engagement, modes of engagement

## Abstract

**Aim:**

Design-based engineering learning (DBEL) offers a potentially valuable approach to engineering education, but its mechanism of action has yet to be verified by empirical studies. Accordingly, the present study aimed to establish whether DBEL produces better learning outcomes, thereby building a strong, empirically grounded case for further research into engineering education.

**Methods:**

To build a more comprehensive model of design-based engineering learning, the variables of cognitive engagement (the mediator) and modes of engagement (the moderator) were introduced to build a theoretical process model. Questionnaires and multiple linear regression analysis were used to verify the model.

**Results and discussion:**

All four features of DBEL (design practice, interactive reflection, knowledge integration, and circular iteration) were found to exert significant and positive effects on learning outcomes. Moreover, cognitive engagement was found to both fully and partially mediate the relationships between these features and the outcomes of engineering learning; under two different modes of engagement, the positive effects of the learning features on cognitive engagement differed significantly.

**Conclusion:**

The paper concluded the following: (1) a design-based learning approach can enhance engineering students’ learning outcomes, (2) cognitive engagement mediates between design-based engineering learning and learning outcomes (3) a systematic mode of engagement produces better learning outcomes than a staged modes of engagement.

## 1. Introduction

The relationship between the theory and practice of modern engineering education has long been a point of debate. Deeply influenced by scientism, the global system of engineering education has shifted increasingly toward the “scientific paradigm” since the 1990s (Fromm, 2003). This paradigm views teacher-student relationships in terms of subjects and objects, thereby shifting the focus of engineering education to the acquisition of formal knowledge. In this paradigm, students gradually become “automatic” knowledge containers that cannot achieve deep learning, higher-order thinking, or conceptual innovation. The National Science Foundation’s Neal Report (1986) emphasized that undergraduate engineering graduates were only equipped with scientific knowledge and tools while lacking practical engineering experience and problem-solving ability (National Science Board, 1986). Another landmark publication entitled “Made in America: Reclaiming the Productivity Edge,” pointed out that U.S. engineering graduates had acquired excellent basic knowledge of engineering science but lacked skills in the areas of critical thinking, comprehensive problem-solving, and teamwork. The report’s authors recommended that modifying current approaches to engineering education would counter the tendency to pursue overly scientific methods of teaching and learning (Dertouzos, 1990). Later, the American Society for Engineering Education (ASEE) proposed that engineering education in the United States should “return to engineering,” integrating scientific theory, professional knowledge, design experience, and engineering practice to build a “big engineering view” (ASEE, 1994; Moses, 1994). Steering engineering education back toward engineering practice has long been an issue for the cultivation of engineering talent.

A great deal of exploratory academic research has led to a wide range of models for delivering effective engineering learning, such as “problem-based” and “project-based” models. These two models are both student-centered, address real-world problems, and emphasize teamwork and exploratory learning (Prideaux, 2003; Hung, 2011). However, project-based learning focuses on completing and presenting physical learning activities (i.e., projects) whereas problem-based learning does not. Neither model focuses on the design element of teaching and learning (National Academy of Engineering (NAE), 2004) even though design is increasingly seen as a key aspect of engineering learning, practice, and innovation (Kolodner et al., 1998; Zou et al., 2013). In Li et al. (2012, p. 2) words, “scientists discover the world as it is, engineers create the world as it has never been”. The function of an engineer is to solve complex industrial problems such as new products, processes, and technologies (Xiang, 2015a,b). Consequently, excluding design from engineering education programs fails to develop students’ intersubjectivity, practical engineering skills, and innovative thinking since it is only through design that practitioners can form a robust architecture of engineering activities into an organic whole (Mitcham, 1994). Overall, the integration of design into learning should be an urgent concern of all stakeholders involved in engineering education.

In the early 21st century, design-based learning (DBL) was introduced to the literature (Doppelt, 2009). In DBL approaches, teachers take a bottom-up approach, posing real-world problems that encourage students to construct meaningful knowledge while completing design tasks. As they work toward a final product that meets task requirements, the students iteratively deepen their theoretical and practical topic knowledge (Goel et al., 1996; Kolodner, 2002a,b; Mehalik and Schunn, 2010; Feiran et al., 2022). DBL is widely viewed as a model that supports innovative learning and has been combined with engineering education practice to evolve into design-based engineering learning (DBEL).

DBEL is a powerful way to induce learning and its conceptual foundations have been studied from various perspectives, including learning processes, teaching tools, and learning models. Some scholars define DBEL as a learning process that is based on authentic engineering design practices and motivates students to construct knowledge while solving engineering problems (Goel et al., 1996; Dym and Shames, 2013; Hadgraft and Kolmos, 2020). Others regard it as a teaching tool for educators to assign challenging tasks and create an interactive environment for students to recall and repeatedly practice what they have learned (Johri and Olds, 2011; Polishetty et al., 2014). DBEL has also been conceptualized as a new approach in which students design specific models using *a priori* experience and learning, cyclically designing and revising these models and acquiring new knowledge in the process (Kimmel et al., 2005; Lee et al., 2010). DBEL has been framed as a collaborative and optimized model of engineering learning in which students continuously analyze and design existing engineering technology systems and improve their quality, functionality, cost, and price to obtain significant improvements in product performance (Polishetty et al., 2014). Finally, it has been argued that DBEL requires students to immerse themselves in challenging situations and design practical, research-based solutions, thereby promoting deeper learning (Kolodner, 2002a,b; Tempelman and Pilot, 2011; Xiang, 2016a,b,c).

Researchers have interpreted DBEL in various ways: first, as *design practice*—a practical process in which engineering students participate in real-world design projects and develop hands-on solutions to actual engineering problems (Gómez et al., 2015; Hadgraft and Kolmos, 2020; Wei, 2022); second, as *interactive reflection*, in which engineering students interact with people and objects as a means of solving design problems (Stevens et al., 2008; Xiang, 2015a,b; Wei, 2022) via frequent and timely reflections and discussions that are constantly summarized to form deeper understandings; third as *knowledge integration*, whereby learners receive external information, integrate this with their internal knowledge and transform both into problem-solving strategies (Watanabe, 1994; Linn et al., 2004; Gomez-del and Rodriguez, 2022; Lina and Wei, 2023); and finally, as *circular iteration*, with students continuously trying out and updating their designs until they verify the overall design solution. These scholars concur that, in DBEL, the repetition of practice is critical because it provides them with the opportunity to test and apply their modified design solutions, explain their experiments, and learn from them (Kolodner, 2002a,b; Dym et al., 2012; Wei, 2022). The four features of design practice, interactive reflection, knowledge integration, and iteration interact with each other to form the dynamic process of design-based engineering learning. Based on the foregoing definitions, this study defines DBEL as a learning mode in which students use their existing knowledge and experience to create physical models that address real-life engineering problems, working through multiple iterations to acquire new knowledge and improve their problem-solving ability.

In the field of engineering education research, scholars have incessantly discerned DBEL, PBL (Project-based Learning) and CDIO (Conceive, Design, Implement, Operate) (Perrenet et al., 2000; Edström and Kolmos, 2014; Zou et al., 2017). All three learning models are based on theories such as constructivism and contextual learning, emphasizing learner-centered construction of knowledge systems around real problems or projects, and thus promoting the development of core competencies of engineering students. The difference between the three is that PBL takes projects as the carrier and emphasizes learners’ mastery of problem-solving methods in project practice, and its evaluation is the quality of students’ project results at a certain stage; DBEL takes design projects, design processes or design courses as the carrier and emphasizes students’ comprehensive design ability to carry out practical exploration in real design tasks. In the research and practice of engineering education at home and abroad, PBL and DBEL are often mutually integrated and embedded in the training programs of engineering students. In terms of scope, CDIO is a form of practical application of DBEL, which helps students actively learn engineering through four major processes of conception, design, realization and operation, and its essential core is consistent with DBEL.

Current research into learning mechanisms has identified the centrality of learning engagement (Kuh, 2003; Betts et al., 2010), whose dimensions include *cognitive engagement*, a key determinant of effective student performance (Meece et al., 1988; Greene and Miller, 1996; Greene, 2015). Cognitive engagement includes the learning strategies that learners take up as they learn and the mental effort they make to acquire knowledge. There are two types of cognitive engagement: the shallow form comprises mechanical memory for information processing, repetitive reading, retelling information, and memorizing new content (Ravindran et al., 2005) whereas deep cognitive engagement includes linking new to old knowledge, building cognitive structures, and applying relevant learning strategies such as planning and self-reflection (Greene and Miller, 1996; Walker et al., 2006). In summary, engineering students enhance cognitive engagement in DBEL, thereby achieving deep learning centered on learner experience.

Looking at the practical cases of engineering education in various countries and regions around the world, there is a general lack of attention to the internal mechanisms and intervention strategies of engineering learning, which poses certain obstacles for students in the process of cultivating paradigms. Due to the lack of relevant theoretical research, the current teaching model lacks theoretical support in the reform of the curriculum system and the optimization of the existing teaching model, and there are bottlenecks and constraints in the reform of design paradigm engineering education. There has been a lack of clear theoretical perspectives and research evidence to examine the relationship between different dimensions of DBEL and engineering learning outcomes.

Hence, the objectives of the present are tripartite: first, to investigate the different dimensions of DBEL (design practice, interactive reflection, knowledge integration and circular iteration); second, to examine the mediating role of cognitive engagement between DBEL and engineering learning outcomes.; and third, to examine the moderating role of modes of engagement between DBEL and cognitive engagement. Therefore, this study has the following research questions. What is the relationship between different dimensions of DBEL and engineering learning outcomes? How cognitive engagement mediates the said relationship? How the modes of engagement moderates DBEL and cognitive engagement.

To achieve these objectives, this study is divided into six parts: the first part proposes the research question, theoretically constructs the process mechanism model of “DBEL → modes of engagement → cognitive engagement → engineering learning outcome,” and puts forward relevant research hypotheses. The second part introduces the data sources and research methods, and develops the measurement scales of relevant variables. The third section presents the results of multiple linear regressions to verify and test the hypotheses. The fourth part further discusses the results of the study. The fifth section gives the conclusions of this paper. The sixth section presents the possible limitations of this paper.

### 1.1. DBEL and engineering learning outcomes

The specific elements of DBEL are difficult to define, and the learning theory that informs it should be elaborated from a perspective that goes beyond specific practices. However, the elements and characteristics of DBEL are yet to be elucidated from such a viewpoint. Scholars at home and abroad generally agree that design-based learning consists of four key dimensions: task, context, activity, and outcome (Wang, 2012; Yang, 2013; Li and Sun, 2015). Taking the components of design-based learning as a logical starting point, there is broad scholarly agreement that design-based engineering learning is dynamically interpreted in students’ design tasks, course instruction, and interactive collaboration.

Scholars argue that the tasks assigned in DBEL models should be authentic, open-ended, challenging, and multidisciplinary (Kimmel et al., 2005; Lee et al., 2010; Zhan and Porter, 2010). On the one hand, such tasks should link teaching scenarios, teacher instruction, and student learning in the classroom with real engineering situations. They should involve teachers and students from different disciplines, thereby encouraging learners to think about needs and solve problems in the role of engineers. On the other hand, the activities themselves should aim to develop specific engineering design skills. Students engaged in DBEL programs should integrate relevant design features into their own engineering tasks and construct their own systems of knowledge as they revise these. In addition, the DBEL model should also include assessments and feedback. These can be process-based measurements of students’ knowledge acquisition and participation, or assessments of the final products of students’ learning (Etkina et al., 2006; Sonia et al., 2013).

**Figure 1:**
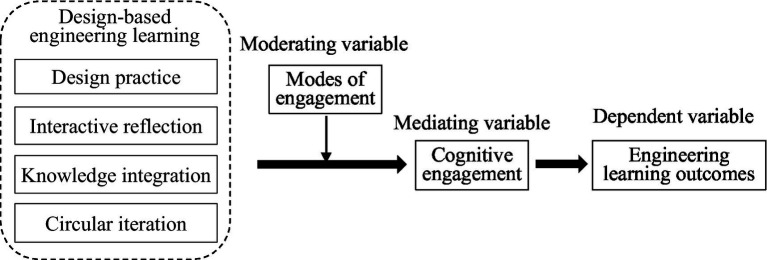
Diagram of the design-based engineering learning research model.

Eisner (1979) introduced the concept of *learning outcome* to denote the result of the learner’s engagement in learning, including not only intentional but also unintentional outcomes. Kuh and Hu (2001) subsequently defined learning outcome as the student’s ability to demonstrate evidence of competence in knowledge, skills, and values after completing a training component or full program. The outcomes of engineering learning programs include the enhancement of subject-specific knowledge, skills, and competencies (Organisation for Economic Co-operation and Development (OECD), 2012; Jia, 2015; Jiang, 2015). DBEL’s direct impacts on the learning outcomes of engineering students have been widely corroborated by researchers (Zhang et al., 2021; Gupta, 2022; Gutierrez-Bucheli et al., 2022). Scholars have pointed out that engineering design activities and tasks center on a cyclic, iterative process of “design–inquiry–redesign,” in which learners’ knowledge and abilities develop in an upward “spiral” pattern (Vincenti, 2001; Xiang, 2015a,b, 2016a,b,c). However, in the field of engineering learning, few empirical studies have examined the relationship between design-based engineering learning and learning effectiveness. To address these issues, a theoretical model of DBEL learning effectiveness was developed (see Figure 1, below). Thus, the initial hypotheses proposed in this study were as follows:

*Hypothesis 1*: DBEL has a positive effect on engineering students’ learning outcomes.

*Hypothesis 1a*: Design practices positively affect engineering students’ learning outcomes.

*Hypothesis 1b*: Interactive reflection positively affects engineering students’ learning outcomes.

*Hypothesis 1c*: Knowledge integration positively affects engineering students’ learning outcomes.

*Hypothesis 1d*: Circular iteration positively affects engineering students’ learning outcomes.

### 1.2. The mediating role of cognitive engagement

In defining the concept of cognitive engagement, most studies have focused on students’ mental activities and cognitive strategies (Fu and Tong, 2016; Ma et al., 2017; Yin and Xu, 2017). For example, Zhu et al. (2009) defined cognitive engagement as the level of mental effort and the type of strategies used by learners as they complete learning tasks, which is related to learning effectiveness; Kong (2000) viewed it as the strategies used by learners engaged in learning processes that induce them to think at different levels. Scholarly work has taken two perspectives on cognitive engagement: one that emphasizes the psychological involvement of learning; and another highlighting the application of learning strategies (Moliterni et al., 1990). Cognitive engagement stems from the perception that learners actively mobilize cognitive, motivational, and emotional aspects when learning, which leads to better outcomes and improves academic performance (Tinto and Pusser, 2006).

Contextual cognitivism views knowledge not as a static intellectual structure confined to the brain, but as a cognitive process that includes people, tools, other people in the environment, and knowledge-building activities (Misra, 2021). Thus, engineering science knowledge is understood as contextual, practical, and produced through collaboration (Brown et al., 1993; Streveler et al., 2008). In a contextual learning model, students will cognitively engage at deeper levels, whereas in a traditional knowledge transfer approach, their cognitive engagement is more superficial. Learners also selectively use deep or surface cognitive engagement according to how they perceive the learning content and context. When classrooms are characterized by clear instructional objectives, sound instructional evaluation, and effective pedagogies, learners tend to adopt deep cognitive engagement and produce better results (Ramsden et al., 2017).

Based on the above analysis, this study anticipated that DBEL would provide an effective contextual learning model in which cognitive engagement plays a crucial mediating role and influences learning outcomes:

*Hypothesis 2*: Different aspects of DBEL positively influence engineering learning outcomes by promoting engineering students’ cognitive engagement.

*Hypothesis 2a*: Design practices positively influence engineering learning outcomes by facilitating the cognitive engagement of engineering students.

*Hypothesis 2b*: Interactive reflection positively influences engineering learning outcomes by facilitating the cognitive engagement of engineering students.

*Hypothesis 2c*: Knowledge integration positively influences engineering learning outcomes by facilitating the cognitive engagement of engineering students.

*Hypothesis 2d*: Circular iteration positively influences engineering learning outcomes by promoting the cognitive engagement of engineering students.

### 1.3. Moderating role of modes of engagement

This study introduces the construct of modes of engagement to characterize design-based engineering learning in different contexts (Lina and Wei, 2023). Based on the literature, these modes of engagement are, in fact, two specific contexts in which students are engaged in design-based engineering learning, labeled here as *staged* and *systematic* engagement. The former refers to the implementation of design-based engineering learning through short-term courses and projects, which often have clear implementation goals, such as a practical project for a particular course or a graduation design. The latter denotes students’ modes of engagement in two or more interrelated design-based engineering learning course modules, which occupy an important place in the four-year undergraduate engineering curriculum.

*Hypothesis 3*: In DBEL, systematic modes of engagement have a stronger positive impact on students’ cognitive engagement than staged modes of engagement.

*Hypothesis 3a*: The positive effect of design practice on the cognitive engagement of engineering students is more significant under systematic modes of engagement.

*Hypothesis 3b*: The positive effect of interactive reflection on the cognitive engagement of engineering students is more significant under the systematic modes of engagement.

*Hypothesis 3c*: The positive effect of knowledge integration on the cognitive engagement of engineering students is more significant under the systematic modes of engagement.

*Hypothesis 3d*: The positive effect of circular iteration on the cognitive engagement of engineering students is more significant under the systematic modes of engagement.

## 2. Materials and methods

### 2.1. Data collection

The data for this study were collected by surveying a sample of engineering students. A total of 2,590 questionnaires were distributed between September 2021 and January 2022, of which 2,210 were returned, a recovery rate of 85.32%. Among these, 560 invalid questionnaires were excluded, leaving 1,650 valid questionnaires, 74.7% of the total and well above the minimum rate specified for this study. All respondents had completed at least one design-based engineering learning project or course. Table 1 summarizes the distribution of valid questionnaires.

**Table 1:** Sample distribution statistics of the questionnaire.

Basic characteristics		Number of samples	Percentage
Types of universities	“985” Universities	759	46.00
	“211” Universities	530	32.12
	General Universities	361	21.88
Gender	Male	1,034	62.67
	Female	616	37.33
Major	Electrical	176	10.67
	Energy and Power	145	8.79
	Electronic Information	176	10.67
	Automation	150	9.09
	Department of Mechanics	78	4.73
	Department of Mechanics	213	12.91
	Computer Science	211	12.79
	Civil Engineering	189	11.45
	Materials	144	8.73
	Chemical and Pharmaceuticals	136	8.24
	Others	32	1.94
Grade	Undergraduate year 1	130	7.88
	Undergraduate year 2	260	15.76
	Undergraduate year 3	498	30.18
	Undergraduate year 4	698	42.30
	Undergraduate year 5	64	3.88
GPA	Top 20% ranking	142	8.61
	Ranking 21–40%	225	13.64
	Ranking 41–60%	400	24.24
	Ranking 61–80%	418	25.33
	Ranking 81–100%	465	28.18

211 University and 985 University are the types of key universities in China, equivalent to the first-class universities recognized by China.

### 2.2. Measures

The main variables measured in this study included engineering learning outcomes (the dependent variable), design-based engineering learning characteristics (the independent variable), cognitive engagement (the mediating variable), and modes of engagement (the moderating variable). The questions used to measure the dependent variable were based on earlier research carried out by Berggren et al. (2003), Kolmos (2011), Pearce and Hadgraft (2011), and Pearce (2015). To measure the multi-dimensional features of DBEL, we referred to studies conducted by Berggren et al. (2003), Kuh (2003), Wang (2018), and Wei (2022) while the measurement questions for the mediating variable were based on work conducted by Stefanou et al. (2013) and Greene (2015). Finally, we referred to Tai et al. (2020) and Wei (2022) to set the measurement questions for the moderating variables. In addition, gender, school, grade, major, and GPA score were included in the regression model as control variables, after previous studies by Zhu (2019), Lian and Shi (2020), and Lv (2020). The questionnaire responses were measured using a 5-point Likert scale, (1 = very non-conforming, 5 = very conforming). The measurement items are shown in Table 2.

**Table 2:** Multidimensional feature measurement items of design-based engineering learning.

Idea	Content of the item	Source of the item
Design practice (DP)	DP1 I learn in real engineering problem situations.	Berggren et al. (2003) and Wei (2022)
	DP2 I learn in explicit design tasks.	
	DP3 I am hands-on, using engineering-related knowledge in problem identification and analysis.	
	DP4 I produce experimental designs to develop, design, and improve systems/components/processes.	
	DP5 I apply techniques and tools to design solutions to engineering problems.	
Interactive reflection (IR)	IR1 I take the initiative to ask questions and participate in discussions with classmates.	Kuh (2003), Wang (2018) and Wei (2022)
	IR2 I form groups with other students for learning and cooperation.	
	IR3 I debate and negotiate problems with my classmates.	
	IR4 I think about solutions to problems from the perspective of others.	
	IR5 I think about the shortcomings of my engineering studies by drawing on a wide range of opinions.	
Knowledge integration (KI)	KI1 I integrate knowledge and methods related to engineering, mathematics, science, or technology courses.	Berggren et al. (2003), Kuh (2003), Wang (2018) and Wei (2022)
	KI2 I combine theory with practice to explore solutions to problems.	
	KI3 I can link processes and synthesize my knowledge.	
	KI4 I can integrate ideas and solutions from peers to generate new ideas.	
Circular iteration (CI)	CI1 I keep trying until I do not repeat the mistakes I’ve made before.	Berggren et al. (2003) and Wei (2022)
	CI2 I adjust my design ideas based on feedback from iterative progress.	
	CI3 I continue to find solutions to problems until I reach my final goal.	
Engineering learning outcomes (ELO)	ELO1 Significant improvement in theoretical engineering knowledge: Extensive knowledge and in-depth understanding and mastery of engineering-related theoretical knowledge, with more emphasis on learning rather than studying.	Berggren et al. (2003), Kolmos (2011), Pearce and Hadgraft (2011) and Jacob and Pearce (2015)
	ELO2 Significant improvement in engineering practical skills: significant improvement of engineering analysis, engineering design, engineering application, and other practical skills.	
	ELO3 Significant improvement in engineering professional skills: a deeper understanding of one’s own profession, and significant improvement of the ability to analyze and solve engineering problems systematically and professionally.	
	ELO4 Significant improvement in engineering innovative thinking: to be able to creatively solve practical engineering problems, and to significantly improve critical thinking, innovation consciousness, and entrepreneurial spirit.	
	ELO5 Significant improvement in engineering communication skills: significant improvement in teamwork, communication and coordination skills, organizational leadership skills, etc.	
	ELO6 Significant improvement in engineering ethics: strong in aspects such as professional ethics, ethical responsibility, and sustainable development.	
	ELO7 Significant improvement in professional identity: having a strong sense of the engineer’s professional identity, and a significant increase in willingness to continue engaging in technical and research work related to this profession.	
Cognitive engagement (CE)	CE1 I look at problems holistically and think about a particular problem from a variety of perspectives.	Stefanou et al. (2013) and Greene (2015)
	CE2 I use diverse learning methods to solve problems.	
	CE3 I analyze and synthesize abstract information to solve practical problems.	
	CE4 I generalize, judge, and compare from different disciplinary directions before reaching an optimal conclusion.	
	CE5 I select, classify, and integrate course materials.	
	CE6 I apply theories or concepts to solve practical problems or apply them to a new situation.	
Modes of engagement (ME)	Systematic engagement is assigned a value of 1 and staged engagement is assigned a value of 0.	Tai et al. (2020) and Wei (2022)

### 2.3. Reliability and validity test

Several tests were used to affirm the reliability and validity of the scales used in this study (see Table 3). First, the Kaiser–Meyer–Olkin (KMO) and Bartlett’s spherical tests were conducted using IBM’s SPSS program (version 26). The KMO values of design-based engineering learning, learning outcomes, and cognitive engagement were 0.982, 0.919, and 0.956, respectively. The Bartlett’s spherical test recorded a significant level, indicating that the data were suitable for factor analysis. On this basis, the factor analysis was validated using AMOS 26.0, which recorded standardized factor loadings higher than 0.8 for all variables. The combined reliability (CR) and Cronbach’s alpha values for all variables were both greater than 0.9, indicating the scale’s high reliability. Meanwhile, the average variance extracted (AVE) values of the variables were all greater than 0.5, verifying the scale’s convergent validity. In addition, the square root of the AVE for each variable was greater than its correlation coefficient, there by indicating the scale’s good discriminant validity.

**Table 3:** Reliability and validity test results.

Variables	Topic term	Factor loadings	Cronbach’s α	KMO	CR	AVE
Design practice	DP1	0.880	0.961	0.982	0.943	0.806
	DP2	0.902				
	DP3	0.910				
	DP4	0.839				
	DP5	0.916				
Interactive reflection	IR1	0.863	0.954			
	IR2	0.905				
	IR3	0.932				
	IR4	0.913				
	IR5	0.905				
Knowledge integration	KI1	0.883	0.973			
	KI2	0.881				
	KI3	0.891				
	KI4	0.905				
Circular iteration	CI1	0.933	0.972			
	CI2	0.929				
	CI3	0.927				
Engineering learning outcomes	ELO1	0.869	0.955	0.919	0.979	0.868
	ELO2	0.886				
	ELO3	0.917				
	ELO4	0.901				
	ELO5	0.855				
	ELO6	0.855				
	ELO7	0.890				
Cognitive engagement	CE1	0.860	0.951	0.956	0.954	0.777
	CE2	0.867				
	CE3	0.865				
	CE4	0.849				
	CE5	0.843				
	CE6	0.847				

### 2.4. Descriptive statistics of the variables

Table 4 displays the means, standard deviations, skewness, kurtosis, and Pearson correlation coefficients of the main variables. The means ranged from 3.06 to 4.02, with standard deviations of between 0.211 and 0.987, and there were positive correlations among the variables. According to Kline (1998), when the absolute value of the skewness of the variables is <3 and the absolute value of the kurtosis is <10, the data follow a normal distribution. In this study, the absolute value of the skewness of all variables in this study did not exceed 0.595 and the absolute value of kurtosis did not exceed 1.668, so the data collected were considered to obey a normal distribution.

**Table 4:** Descriptive statistics of the main variables measured by the formal questionnaire.

Variables	DP	IR	KI	CI	CE	ELO	PM
DP	1						
IR	0.533**	1					
KI	0.542**	0.505**	1				
CI	0.500**	0.517**	0.536**	1			
CE	0.062*	0.061*	0.041*	0.134**	1		
ELO	0.525**	0.544**	0.530**	0.592**	0.144**	1	
PM	0.139**	0.151**	0.234**	0.147**	0.139**	0.121**	1
Mean value	3.98	4.02	3.87	3.84	3.06	3.79	0.41
Standard deviation	0.845	0.867	0.961	0.987	0.211	0.722	0.392

**p ≤ 0.01 (bilateral); *p ≤ 0.05 (bilateral).

## 3. Results

### 3.1. Effect of design-based engineering learning on engineering learning outcomes

Table 5 reports the regression results for the linkages between various features of DBEL and engineering learning outcomes. The results show that design practice had a significant positive effect on these outcomes (*β* = 0.365, *p* < 0.001), as did interactive reflection (*β* = 0.103, *p* < 0.001), knowledge integration (*β* = 0.198, *p* < 0.001), and circular iteration (*β* = 0.313, *p* < 0.001). Therefore, *Hypotheses 1a*, *1b*, *1c*, and *1d* were supported.

**Table 5:** Regression analysis of the effect of DBEL on engineering learning outcomes.

	Dependent variable: engineering learning outcomes				
	Model 1	Model 2	Model 3	Model 4	Model 5
Gender	−0.004	0.005	0.008	0.010	0.006
Grade	0.037	0.012	0.003	−0.025*	−0.022*
Types of universities	0.122***	0.062***	0.044***	0.033**	0.024*
Major	0.018	−0.026	−0.015	−0.010	−0.010
GPA	0.134***	−0.006	−0.010	−0.037*	−0.001
Design practice		0.831***	0.437***	0.390***	0.365***
Interactive reflection			0.498***	0.348***	0.103***
Knowledge integration				0.228***	0.198***
Circular iteration					0.313***
Adjusted *R*^2^	0.036	0.669	0.779	0.792	0.805
*F*-value	13.481	616.541	594.667	104.192	113.109
VIF value	1.070–1.197	1.070–1.199	1.070–2.372	1.071–2.482	1.071–3.072
VIF average value	1.109	1.105	1.465	1.720	1.960

*p < 0.05; **p < 0.01; ***p < 0.001.

### 3.2. Mediating effects of cognitive engagement

#### 3.2.1. Test for mediating effects of cognitive engagements

The overall theoretical model suggested that cognitive engagement might mediate between design practices, cyclical iterations and interactive reflections, and learning outcomes. To decide how to test these hypothesized relationships, we consulted related studies such as Baron and Kenny (1986), Jiang (2022), Wen et al. (2022), and Fang et al. (2023). Stepwise regression and bootstrapping were used to test the mediating effect of cognitive engagement.

Model 6 showed that design practice, interactive reflection, knowledge integration, and circular iteration imparted a significant positive effect on the cognitive engagement of the engineering students (see Table 6) while model 9 demonstrated that cognitive engagement had a significant positive effect on learning outcomes. Comparing models 8 and 9, it was noted that the coefficients of design practice, knowledge integration, and circular iteration with engineering students’ learning outcomes changed significantly after the mediating variable of cognitive engagement was introduced while the effect of interactive reflection on the engineering students’ learning outcomes became insignificant.

**Table 6:** Test of the mediating effects of cognitive engagement on the relationship between multidimensional learning features and engineering learning outcomes.

	Dependent variable: cognitive engagement	Dependent variable: engineering learning outcomes		
	Mode 6	Mode 7	Mode 8	Mode 9
Gender	−0.024	−0.004	0.006	0.016
Grade	−0.026*	0.037	−0.022*	−0.011
Types of universities	0.011	0.122***	0.024*	0.019
Major	0.000	0.018	−0.010	−0.010
GPA	0.046***	0.134***	−0.001	−0.017
Design practice	0.362***		0.365***	0.214***
Interactive reflection	0.116***		0.186***	0.022
Knowledge integration	0.193***		0.198***	0.150***
Circular iteration	0.308***		0.313***	0.184***
Cognitive engagement				0.419***
Adjusted *R*^2^	0.828	0.016	0.047	0.053
F-value	123.744	5.238	13.439	14.131
VIF value	1.071–3.072	1.070–1.152	1.071–3.072	1.019–3.190
VIF average value	1.960	1.12875	1.960	1.963

*p < 0.05; **p < 0.01; ***p < 0.001.

In summary, as Table 6 demonstrates, cognitive engagement mediated each of the relationships between design practice/knowledge integration/circular iteration and engineering students’ learning outcomes, thereby verifying *Hypotheses 2a*, *2c*, and *2d*. Moreover, it fully mediated the path between interactive reflection and learning outcomes, thereby supporting *Hypothesis 2b*.

#### 3.2.2. Bootstrap test analysis for the significance of the mediating effect

Based on the preliminary results, basic bootstrap resampling was conducted using Stata16 software to empirically analyze the mediating effects of cognitive engagements. In this study, 2000 bootstrap resampling analyses were conducted based on the 1,650 samples to obtain the standard deviation, significance, and 95% confidence intervals of the direct, indirect, and total effect unstandardized path coefficients of the model path analysis. The test results are shown in Table 7.

**Table 7:** Results of the analysis of the bootstrap test for the significance of mediation effects.

Intermediary model	Total effect	Direct effect	Indirect effect [95%, CI]
DBEL→CE → ELO	0.882***	0.477***	0.405*** [0.317, 0.503]
DP → CE → ELO	0.749***	0.242***	0.509*** [0.448, 0.565]
IR → CE → ELO	0.787***	0.269***	0.517*** [0.437, 0.594]
KI → CE → ELO	0.668***	0.206***	0.462*** [0.406, 0.517]
CI → CE → ELO	0.790***	0.289***	0.501*** [0.422, 0.583]

*p < 0.05; **p < 0.01; ***p < 0.001 (*N* = 1,650).

The investigation of the mediating role of cognitive engagement showed that its mediation of the relationship between design-based engineering learning and engineering learning outcomes was significant, with an indirect effect value of 0.405 (*p* < 0.001) and a 95% confidence interval of [0.317, 0.503]. Cognitive engagement also significantly mediated the effects of the following aspects of DBEL on engineering learning outcomes: design practice (0.509, *p* < 0.001, 95% CI [0.448, 0.565]), interactive reflection (0.517, *p* < 0.001, 95% CI [0.437, 0.594]), knowledge integration (0.462, *p* < 0.001, 95% CI [0.406, 0.517]), and circular iteration (0.501 *p* < 0.001, 95% CI [0.422, 0.583]). In summary, *Hypotheses 2a*, *2b*, *2c*, and *2d* were tested and all four were verified.

### 3.3. Moderating effect of modes of engagement

Following Fang et al. (2022), group regression and interaction terms were then used to test the moderating effect of modes of engagement. The sample was divided into two groups according to the type of modes of engagement (systematic vs. staged), and group regressions were randomly conducted using SPSS (see Table 8).

**Table 8:** The moderating effects of modes of engagement and cognitive engagement in DBEL.

Dependent variable: cognitive engagement	Model 10	Model 11	Model 12
	Staged engagement	Systematic engagement	
Gender	0.032	0.022	0.006
Grade	−0.023	−0.045	−0.022
Types of universities	0.023	0.012	0.021
Major	−0.054	0.005	−0.010
GPA	0.017	0.017	−0.010
Design practice (DP)	0.159**	0.450***	0.368***
Interactive reflection (IR)	0.097*	0.104*	0.190***
Knowledge integration (KI)	0.152	0.115*	0.117***
Circular iteration (CI)	0.587***	0.308***	0.306***
DP × PM			0.049*
IR × PM			0.081**
KI × PM			0.005
CI × PM			0.024
Adjusted *R*^2^	0.772	0.871	0.906
F-value	218.262***	388.540***	577.597***
VIF value	1.070–3.123	1.070–2.868	1.070–2.973
VIF average value	1.967	1.816	1.903

*p < 0.05; **p < 0.01; ***p < 0.001.

In model 10 (systematic engagement in design-based learning), design practice, interactive reflection, knowledge integration, and circular iteration had significant positive effects on engineering students’ learning outcomes. However, in model 11 (the staged engagement model), only the first three of these had significant positive effects on learning outcomes while the effect of knowledge integration was insignificant.

Finally, the systematic and staged engagement modes were set to 0 and 1, respectively and their interactions with design practice, interactive reflection, knowledge integration, and cyclic iteration were tested. The results showed positive and significant interaction terms for the mode of engagement and the two variables of design practice (*β* = 0.049, *p* < 0.05) and interactive reflection (*β* = 0.081, *p* < 0.001). However, the corresponding terms for knowledge integration and circular iteration were not significant (*β* = 0.005, *p* > 0.05; *β* = 0.024, *p* > 0.05). These results therefore supported *Hypotheses 3a* and *3b* but did not verify *3c* and *3d*.

## 4. Discussion

### 4.1. Design-based engineering learning effectively enhances engineering students’ learning outcomes

This study empirically tested the significant positive effects of four learning characteristics on learning outcomes through multiple regression analysis. First, the test results showed a significant positive effect of design practices on engineering students’ learning outcomes, thereby supporting *Hypothesis 1a*. Task-specific problem situations appear to stimulate learners’ engagement, in turn improving their learning outcomes. The findings of this study affirmed the important role of design practices in enhancing engineering students’ learning outcomes and believed that specific learning tasks could help deconstruct complex knowledge systems and enhance learners’ cognitive engagement, to some extent.

Second, interactive reflection significantly and positively affected engineering students’ learning outcomes, thus supporting *Hypothesis 1b*. There are two reasons why interactive reflection improves engineering students’ learning outcomes: first, interactive reflection offers a crucial way for learners to communicate with the outside world and transform the information they gain into their own knowledge; second, interactive reflection can construct a discourse of mutual understanding and facilitate the application and implementation of technology.

Third, the empirical test results show that knowledge integration exerted a positive effect on engineering students’ learning outcomes, confirming *Hypothesis 1c*. Knowledge integration demonstrates learners’ ability to coordinate and integrate key resources. It also enables the smooth flow of scientific thinking and disciplinary knowledge across boundaries, promotes efficient communication within organizations, and enhances the learning outcomes of engineering students.

Fourth, circular iteration was found to positively affect the learning outcomes of engineering students, thus verifying *Hypothesis 1d*. In student-centered engineering, circular iteration may gradually be marginalized with students’ initiative and motivation assuming greater prominence in pedagogy.

In recent years, the importance of teamwork for learning quality improvement and core competency development has been demonstrated in this study and in numerous studies on collaborative learning (Collazos et al., 2007; Collins, 2008; Nguyen et al., 2020), especially since engineering learning and engineering work are socio-technical in nature and require a greater focus on collaboration with others (Brunhaver et al., 2017). DBEL often has a clear learning theme, and students are encouraged to organize collaborative learning teams around a certain project design task or theme, with team members working together to complete one or more real engineering design practice tasks, it will continuously strengthen the cultural atmosphere of engineering practice in teams and students’ sense of engineering ethics and responsibility. These activities will improve the engineering professional identity of team members, especially to carry out effective engineering practice in the social framework and values in which engineering students live, and effectively promote the quality improvement of engineering learning.

### 4.2. Cognitive engagement mediates the relationship between DBEL and learning outcomes

The test of mediating effects revealed that cognitive engagement partially mediated the relationships between design practice, knowledge integration, circular iteration, and engineering students’ learning outcomes while fully mediating the link between interactive reflection and learning outcomes. These results were further confirmed by bootstrap resampling, demonstrating that cognitive engagement was an important mediator of the DBEL mechanism and enhanced engineering students’ learning outcomes. Relevant research also supports this conclusion (Guo and Ji, 2019).

For engineering students, cognitive engagement is more reflected in their engineering learning experiences and feelings. Therefore, learning experience is very important for engineering students. Generally speaking, the application scenarios of DBEL mainly include single-course or project-based teaching, challenging tasks, etc. It requires the students to play the main position in the design experience, and improves the adaptability of course-practice integration through the community-based project context to run through the course and practice modules, which helps students enhance the meaning of engineering knowledge and understanding in the interaction between engineering knowledge learning and real engineering design practice. And thus improve their learning experience and cognitive engagement. However, how to enhance students’ learning experience in non-design courses has long been an important and difficult question to answer, especially when the COVID-19 pandemic has forced global education systems to adapt to online learning. To this end, we can try to use virtual reality and other internet technologies to transform practical training application scenes into practical teaching image materials, break through the actual space scenes and form a unique online teaching mode.

### 4.3. Modes of engagement moderates the relationship between DBEL and cognitive engagement

Modes of engagement were found to significantly moderate the relationship between DBEL and cognitive engagement. In DBEL, a systematic mode of engagement was more likely to enhance engineering students’ cognitive engagement than one that is stage-based, thereby promoting their learning outcomes. One possible explanation for this influence is related to the interrelated course modules provided by the system participation model throughout the entire process of engineering learning. In this context, students can participate in more systematic design courses and may have more opportunities to try various engineering design experiences (Kong, 2000).

This provides a basis for universities to prioritize organizational support for DBEL. Therefore, universities should combine different learning contexts and organizational characteristics to flexibly embed DBEL into undergraduate engineering education and improve the learning effect of engineering students comprehensively. Universities can make use of staged engagement model to flexibly apply DBEL in independent or integrated teaching design and project practice; and make use of systematic engagement model to fully apply DBEL in the construction of core professional modules for engineering students. At the same time, universities can make full use of the practical training system combining experiment, simulation, internship and graduation design throughout the four-year university training plan of engineering majors to realize the cultivation of students’ engineering design thinking ability.

## 5. Conclusion

In conclusion, this study used a large sample to empirically test the effects of four design-based learning characteristics of engineering education on student learning outcomes. Its in-depth investigation of the characteristics of DBEL and their mechanisms of action has addressed several limitations of existing theories. Our holistic framework connects the key aspects of design-based engineering learning to modes of engagement, cognitive engagement, and engineering learning outcomes (see Figure 1). Taking a dynamic perspective, we focused on the characteristics of DBEL in colleges and universities and analyzed its mechanism of effect in more detail. By proposing and rigorously testing a model of DBEL, we have extended the boundaries of research into engineering learning and revealed the systematic correlations among the features of engineering learning under the design paradigm, thereby providing a conceptual and empirical basis for the model. The research establishes an empirical basis for reforming and implementing a design-based engineering learning model in colleges and universities. By examining two different modes of engagement, we show that systematic design-based programs of engineering learning in colleges and universities can improve students’ learning outcomes. The study highlights the need for colleges and universities to address the institutional and cultural barriers to providing adequate support for DBEL.

## 6. Limitations and prospects

This empirical study has several shortcomings. The distribution of the sample may not be fully balanced since, among the 1,650 engineering undergraduates who returned valid responses, 46% were from 985 universities, 32.12% were from 211 universities, and 21.88% were from ordinary undergraduate universities. Different universities have different educational resources and students’ quality, which may affect the implementation effect of DBEL. Future studies should investigate the effects of institution type on the different dimensions of engineering students’ learning performance, as well as any variations that occur according to modes of engagement.

## Data availability statement

The raw data supporting the conclusions of this article will be made available by the authors, without undue reservation.

## Author contributions

LW designed the study, collected the data, and drafted the manuscript. WZ contributed ideas to the manuscript. CL was responsible for revising the article. All authors contributed to the article and approved the submitted version.

## Funding

This study was supported by the National Natural Science Foundation of China program (72074191), the Zhejiang Provincial Natural Science Foundation program (LZ22G030004), and the Chinese Society of Academic Degrees and Graduate Education program (2020ZD1014).

## Conflict of interest

The authors declare that the research was conducted in the absence of any commercial or financial relationships that could be construed as a potential conflict of interest.

## Publisher’s note

All claims expressed in this article are solely those of the authors and do not necessarily represent those of their affiliated organizations, or those of the publisher, the editors and the reviewers. Any product that may be evaluated in this article, or claim that may be made by its manufacturer, is not guaranteed or endorsed by the publisher.
